# The Effect of Plasma Protein Binding on the Therapeutic Monitoring of Antiseizure Medications

**DOI:** 10.3390/pharmaceutics13081208

**Published:** 2021-08-05

**Authors:** Bruno Charlier, Albino Coglianese, Federica De Rosa, Ugo de Grazia, Francesca Felicia Operto, Giangennaro Coppola, Amelia Filippelli, Fabrizio Dal Piaz, Viviana Izzo

**Affiliations:** 1 Department of Medicine, Surgery and Dentistry “Scuola Medica Salernitana”, University of Salerno, 84081 Baronissi, Italy; bcharlier@unisa.it (B.C.); acoglianese@unisa.it (A.C.); federica.derosa86@gmail.com (F.D.R.); gcoppola@unisa.it (G.C.); afilippelli@unisa.it (A.F.); fdalpiaz@unisa.it (F.D.P.); 2 Operative Unit of Clinical Pharmacology, University Hospital “San Giovanni di Dio e Ruggi d’Aragona”, 84131 Salerno, Italy; 3 Laboratory of Neurological Biochemistry and Neuropharmacology, Fondazione IRCCS “Istituto Neurologico Carlo Besta”, 20133 Milano, Italy; ugo.degrazia@istituto-besta.it; 4 Operative Unit of Child Neuropsychiatry, University Hospital “San Giovanni di Dio e Ruggi d’Aragona”, 84131 Salerno, Italy; opertofancesca@gmail.com

**Keywords:** antiseizure medications, antiepileptic drugs, LC-MS/MS, pharmacokinetics, therapeutic drug monitoring

## Abstract

Epilepsy is a widely diffused neurological disorder including a heterogeneous range of syndromes with different aetiology, severity and prognosis. Pharmacological treatments are based on the use, either in mono- or in polytherapy, of antiseizure medications (ASMs), which act at different synaptic levels, generally modifying the excitatory and/or inhibitory response through different action mechanisms. To reduce the risk of adverse effects and drug interactions, ASMs levels should be closely evaluated in biological fluids performing an appropriate Therapeutic Drug Monitoring (TDM). However, many decisions in TDM are based on the determination of the total drug concentration although measurement of the free fraction, which is not bound to plasma proteins, is becoming of ever-increasing importance since it correlates better with pharmacological and toxicological effects. Aim of this work has been to review methodological aspects concerning the evaluation of the free plasmatic fraction of some ASMs, focusing on the effect and the clinical significance that drug-protein binding has in the case of widely used drugs such as valproic acid, phenytoin, perampanel and carbamazepine. Although several validated methodologies are currently available which are effective in separating and quantifying the different forms of a drug, prospective validation studies are undoubtedly needed to better correlate, in real-world clinical contexts, pharmacokinetic monitoring to clinical outcomes.

## 1. Introduction

Epilepsy is a widely diffused neurological disorder affecting approximately 70 million people worldwide, which includes a heterogeneous range of syndromes with different aetiology, severity, and prognosis [1]. There are various treatments aimed at controlling the different types of seizures, most of which include the use, either in mono- or in polytherapy, of drugs acting on the epileptic symptoms. Approaches such as neuromodulation, neurosurgical interventions, or ketogenic diet are less common because they are either too invasive or still under debate in terms of efficacy [2,3,4].

**Figure 1:**
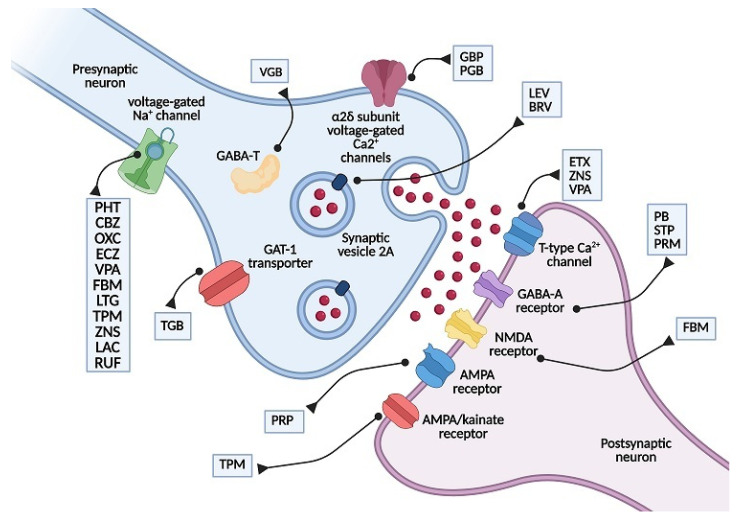
Mechanism of action of commonly used ASMs (Created by BioRender.com).

Antiseizure medications (ASMs) [5] act at different synaptic levels, generally modifying the excitatory and/or inhibitory response through different mechanisms. Some ASMs have an effect on the neurotransmission of γ-aminobutyric acid (GABA); as an example, phenobarbital (PB) and stiripentol (STP) bind to the GABA-A receptor causing a prolongation of the chloride associated channel opening, thus determining an improved inhibitory activity of the neurotransmitter [6]. On the other hand, vigabatrin (VGB) reversibly binds GABA transaminase enzyme (GABA-T), which hampers GABA degradation and increases its concentration in the brain, while tiagabine (TGB) inhibits GABA reuptake by blocking the GAT-1 transporter [7]. A great number of ASMs interact with ion channels, preventing ion flow and altering the neuronal action potential. Phenytoin (PHT), carbamazepine (CBZ), oxcarbazepine (OXC), eslicarbazepine (ECZ), valproic acid (VPA), felbamate (FBM), lamotrigine (LTG), topiramate (TPM), zonisamide (ZNS), lacosamide (LAC), and rufinamide (RUF) bind to voltage gated sodium channels, stabilizing them in an inactive configuration and reducing high-frequency neuronal activation [8]. Ethosuximide (ETX) and ZNS exert their action on T-type calcium channels, regulating neuronal excitability at the thalamus level [9]. Gabapentin (GBP) and pregabalin (PGB) binds to the α-2-δ subunit of voltage-gated calcium channels, reducing calcium influx and blocking neurotransmitter release [10]. Perampanel (PRP) is a selective non-competitive α-amino-3-hydroxy-5-methylisoxazole-4-propionic (AMPA) glutamate receptor antagonist [11]. Levetiracetam (LEV) and brivaracetam (BRV) bind to synaptic vesicle protein SV2A, which seems to decrease neurotransmitter release in a state of neuronal hyperactivation [12]. Besides, some ASMs have multiple mechanisms of action: FBM, in addition to blocking sodium channels, is also an N-methyl-D-aspartate (NMDA) receptor antagonist; TPM is also an AMPA/kainate receptor antagonist and increases GABA activity; VPA and ZNS hinder both T-type calcium and sodium channel openings, and VPA also increases GABA activity [9,13] (Figure 1).

ASMs authorized for distribution from the early years of the last century (PB from 1912) up to 1970 may be classified as “first generation”. These molecules are characterized by several disadvantages, such as strong protein-binding and either inhibition or induction of metabolic pathways (See Table 1), drawbacks that following generations of drugs have tried to overcome. Second-generation ASMs have been approved since 1980, while newer ASMs approved after 2008 are referred to as third-generation drugs [1,14]. First- and second-generation ASMs generally display a narrow therapeutic index that, combined with significant interindividual pharmacokinetic variability (including absorption, distribution, metabolism, and excretion), is responsible for well-known toxicity problems [15]. Compared to the first- and second-generation, third-generation ASMs show better bioavailability, decreased binding to plasma proteins (except PRP), and fewer drug–drug interactions [16].

To reduce the risk of adverse effects and drug interactions, therapeutic drug monitoring (TDM) to evaluate plasma levels of ASMs is strongly advisable [17,18]. This clinical practice, which involves the measurement of ASMs concentration at designated time intervals to maintain a constant value in the patient bloodstream, helps to prevent seizures, minimize adverse effects, and optimize individual dosage regimens [18,19]. Among others, dosage of ASMs plasma concentration is of utmost importance in paediatric patients, which undergo marked hormonal and neurobiological changes during their development and often require adjustments of dosage regimens. Indeed, drugs pharmacokinetics widely vary in children with epilepsy due to age-related factors, which can influence absorption, distribution, metabolism, and elimination of the pharmacological agent [20,21].

The pharmacological targets of ASMs are all located in the Central Nervous System (CNS), therefore these drugs should be able to move across the blood–brain barrier (BBB), a highly selective barrier formed by endothelial cells connected by tight junctions separating circulating blood from brain extracellular fluid [22]. Tight junctions between cells and microvessels hamper macromolecules and hydrophilic fluids from crossing the BBB by simple diffusion, and permeability is only granted to small (<500 Da), lipophilic, and uncharged compounds [22]. There are three potential BBB drug-crossing mechanisms: small, lipophilic, and uncharged ASMs can easily access the CNS by passive diffusion [23]; ASMs endogenous carriers mediate small water-soluble ASMs transport; polar or higher molecular weight ASMs use a receptor-mediated transport [24]. As an example, VPA uses monocarboxylate transporter [25,26], while Gabapentin crosses the BBB using the amino acid transporter [24]. Consequently, the measurement of ASMs blood concentration alone is generally not sufficient to evaluate their actual bioavailability, which is strongly influenced by their hydrophilicity and affinity for plasma proteins [17]. Indeed, only the drug fraction that is not bound to proteins (the so-called “free” drug) may cross the BBB and reach its molecular target.

Although several ASMs display linear kinetics and low protein binding, some new generation drugs, such as PRP, and most of the older ASMs (i.e., including PHT, VPA, TGB, and STB) strongly interact with albumin and α1 acid glycoprotein [27] and their protein-bound fraction can be higher than 90%. This may lead to significant interactions for the patients undergoing polytherapy, due to the potential displacement of concomitant drugs bound to plasma proteins and the unexpected increase in the free drug fractions. The latter may be higher than expected also in pathological conditions including uremia, liver disease, and hypoalbuminemia. For example, VPA and PHT display 90% binding to plasma proteins when given singularly; this fraction can be significantly reduced in the case of hypoalbuminemia or when other highly protein-bound drugs are co-administered, leading to an increase in the free fraction amounts and, potentially, dose-dependent adverse reactions and toxic effects.

The measurement of drug levels in biological samples can be given as either total concentration, which ignores drug interaction with the sample matrix, or as free concentration, which instead gives an indication of the effective amount of drug capable of spreading across membranes and exerting its biological activity. Many decisions in TDM are based on the determination of the total drug concentration, although measurement of the free fraction is becoming in many situations increasingly important to shed light on observed pharmacological and toxicological effects. When the relationship between the bound and free portion of the drug is constant, the evaluation of its total concentration may be considered satisfactory. However, in situations involving the use of highly bound ASMs such as PHT, VPA, TGB, and PRP, free drug concentrations are significantly higher than the prediction based on total drug concentration [17,27,28,29]. In such cases, the evaluation of total concentration would be confounding and only the measurement of free drug serum concentration would actually be related to drug toxicity and efficacy (Table 1).

The aim of this work is to review the methodological aspects concerning the evaluation of the free plasmatic fraction of some ASMs, focusing attention on the effect that drug–protein binding has in the case of widely used drugs such as VPA, PHT, PRP, and CBZ.

## 2. Analytical Tools for the Evaluation of ASMs’ Free Drug Fraction

From an analytical point of view, methods for measuring total drug concentrations require less time and resources than those aimed at quantifying the free drug, which often involves complex sample preparation procedures and more sensitive analytical tools [37,38].

**Figure 2:**
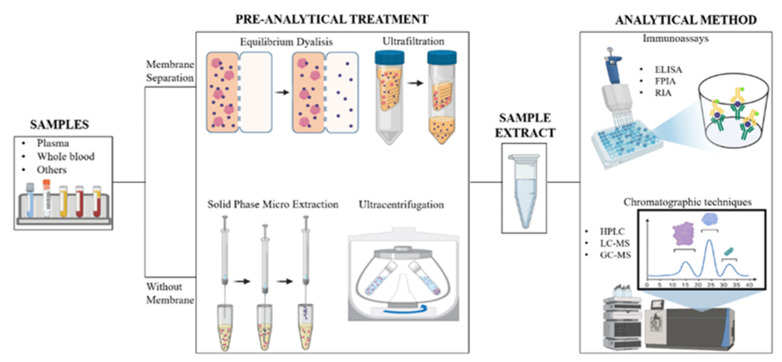
Schematic representation of the workflow carried out for the quantization of free and protein-bound drug in a biological matrix (Image created with BioRender.com).

Free drug analysis involves the separation of bound and unbound drug, achieved through various methods briefly summarized in this paragraph, followed by an appropriate detection and quantification approach; this latter is selected considering molecules’ chemical nature and the type of matrix analyzed, and mainly involves chromatography or immunoassays [39] (Figure 2).

Immunoassays are generally faster than chromatographic techniques, but need costly reagents, longer sample preparation times, and sometimes analyte derivatization [40]. In addition, they can suffer from interferences and cross-reactivity due to the presence of compounds similar to the investigated drugs or metabolites [41]. The main immunoassays used to quantify ASMs include radioimmunoassays (RIAs), enzyme-linked immunosorbent assays (ELISAs), and fluorescence polarization immunoassays (FPIAs) [42]. For first-generation ASMs, available commercial kits are based on competitive homogenous immunoassays performed using routine automated analyzers, frequently present in hospital settings [15]. Until the past decade, the vast majority of TDM analyses on these drugs was performed using these approaches. Indeed, in December 2011 the College of American Pathologists Proficiency Survey (CAPPS) declared that among 125 participating laboratories that performed free PHT assay, none of them used chromatographic techniques. The most used method was the FPIA assay on TDx analyzer (Abbott, Chicago, IL), which is based on a fluorescence polarization homogeneous competitive immunoassay involving the use of a labeled drug that competes with free PHT for antibody binding sites. However, when the Abbott TDx FPIA was retired from the market, many laboratories were forced to subordinate the analyses to an external reference laboratory or to adapt a total PHT assay to measure the free fraction. Dasgupta and coworkers succeeded in optimizing the use of free PHT calibrators of the Cobas Integra analyzer (Roche Diagnostics Indianapolis, IN), on the Cobas c501 analyzer designed for total drug determination and validated their assay on TDx analyzer with comparable results [43]. Another similar example, described by Williams et al., involved an in-house development of a bidentate turbidimetric inhibition FP immunoassay, employing reagents already in use for total PHT measurements (DxC800 Beckman Coulter, Brea, CA). They validated their method on 97 patients and compared the results with those obtained using a TDx analyzer, obtaining a satisfying correlation between the two methods [44]. It is, however, worth remembering that immunoassays allow quantifying a single compound (drug or metabolite) at a time. Furthermore, only a limited number of ASMs are measurable using these techniques [45], depending on the availability of the required specific antibody.

As an alternative, chromatography-based methods coupled with different detectors may be used, as they provide physical separation of serum components before the analysis. Chromatographic techniques are now considered the gold standard in TDM for their specificity and robustness, although they require trained personnel, specialized laboratories, and long sample processing times [45,46,47,48,49,50]. Several reported procedures in TDM use liquid chromatography coupled with tandem mass spectrometry (LC-MS/MS), which can discriminate similar compounds based on their retention times, *m*/*z* values of the respective pseudo molecular ion, and fragmentation patterns, thus ensuring great specificity in the identification of the analytes [51,52,53,54]. Since the 2000s, the LC-MS/MS methods were largely adopted for the quantification of free drug concentrations in biological matrices, mainly in plasma and interstitial fluids, providing sensitive detection and accurate results [52,53].

Simultaneous quantification of chemically similar molecules may be challenging, due to comparable chromatographic behavior, variable ionization efficiency, and overlapping *m/z* values of precursor and product ions [41]. Although several papers describe the use of LC-MS methods for the identification and quantification of many ASMs within a single analytical run, most of them have focused their attention on the evaluation of their total amount, regardless of their binding to plasma proteins [53,54,55]. An example reporting a complete evaluation of ASMs plasma levels is given by Patsalos and coworkers, who developed an LC-MS method to measure both free and total concentration of 25 different ASMs in a single run [27]. Conversely, many methods were set up to measure the free fraction of a single drug; in particular, most of them are focused on the analysis of PHT and VPA by HPLC-UV, LC-MS/MS [56,57,58], or GC techniques [59].

Regardless of the analytical technique used, the evaluation of the drug free fraction mostly depends on the type of pre-analytical technique used to separate the bound and unbound drug. Over the past few decades, many methodologies have been developed for determining the extent of plasma protein binding of drugs. In the clinical evaluation of drug therapy, equilibrium dialysis (ED), ultrafiltration (UF), and ultracentrifugation (UC) are the most routinely utilized techniques [60], although they all suffer from equilibrium disturbance and nonspecific adsorption (NSA) [61] (Figure 2).

UF methodologies use semipermeable membranes with a specific molecular weight cut-off, which allow retaining proteins from the filtered sample. When a drug-containing plasma sample is placed in the UF apparatus, plasma proteins and all the compounds bound to them are retained; small molecules and unbound drugs pass through the filter membrane after a soft centrifugation [62]. The UF approach is fast, simple, and can be used to process many samples simultaneously. It is applicable to different types of biological matrices, including tissue homogenates [60]. The major limitation of the UF technique, however, is NSA to filter membranes [63]. Drug lipophilicity and dimensions are the main parameters that influence NSA. Indeed, when a strong interaction between membrane and drug occurs, the concentration of the free fraction determined differs significantly from the actual one, leading to inaccurate results [64].

ED is the most frequently used methodology to evaluate plasma protein binding in basic research. The apparatus used in this method consists of two chambers separated by a high-molecular-weight cutoff membrane, available with various molecular weight cut-offs. The free-drug fraction diffuses across the membrane, and its concentration in the two chambers reaches the equilibrium after several hours. Afterwards, the solution present in each reservoir is removed and analyzed to measure the drug concentration [39]. ED is often considered the “gold standard” [39,62,65], but there are several drawbacks associated with the use of this technique [61]. First, it takes a long time to reach equilibrium, from a minimum of four to a maximum of 28 h. Moreover, since the sample is incubated at physiological temperature and pH, the stability of the drug and of the protein/drug complex at 37 °C and pH 7.4 should be carefully evaluated [39,61]. Despite these limits, ED is still considered a reference method and NSA is believed not to affect the evaluation of the unbound fraction [60,61,62]. Unfortunately, this technique is undoubtedly time consuming and not suitable for routine clinical trials or hospital laboratories; therefore, some authors are considering validating new methodological approaches [60].

Recently, hollow fiber centrifugal ultrafiltration (HFCF-UF) has been introduced for the separation of unbound drug fraction from plasma samples. This technique includes a simple device composed of a slim glass tube and a U-shaped hollow fiber [66]. Zhang and coworkers compared this methodology to traditional UF and demonstrated that HFCF-UF is not dependent on ultrafiltrate volume for accurate measurement of plasma unbound drug. In this approach, indeed, ultrafiltrate volume can be easily controlled by the hollow fiber inner diameter without the need for further time-consuming operations [67]. The same authors also investigated the effect of plasma albumin concentration on unbound ASMs measurement [68]. In this study, VPA was used as a reference drug, and plasma samples with different albumin concentrations were analyzed by UF and HFCF-UF. Quantitative analysis on the resulting samples was then performed using an HPLC system coupled with a UV detector. The method displayed good sensitivity and accuracy, a low limit of detection and quantification (0.01 and 0.05 µg/mL, respectively), and good linearity in the concentration range of 0.05–200 µg/mL for free VPA. At high albumin concentrations (40 and 60 g/L), drug free concentrations measured by HFCF-UF and UF were similar, but a significant difference was observed in the results obtained at low albumin concentrations (<40 g/L), and this difference increased following albumin concentration decrease. These results suggested that accuracy measurement of free VPA using traditional UF pre-treatment was dependent on albumin concentration; therefore, the authors recommend that the HFCF-UF should be the method of choice for the TDM of plasma free VPA [68]. In another work, Gao and coworkers applied HFCF-UF coupled to HPLC to quantify free CBZ in clinical samples [69]. The main advantage of this procedure was the possibility of evaluating both free and total concentrations from the same plasma sample. In this work, 500 µL of plasma were subjected to HFCF-UF and, after centrifugation, free CBZ was obtained from the ultrafiltrate, recovered from the hollow fiber, and directly injected into the HPLC system. Conversely, the bound drug portion was obtained by solvent extraction on the concentrated plasma sample using the same HFCF-UF device. The authors reported high recovery and reproducibility and no significant NSA. The method developed was successfully applied in the TDM of 20 epileptic patients undergoing CBZ therapy, but further studies on a larger number of patients are undoubtedly required to fully evaluate the potential of this approach [69].

Promising results were also achieved using the classical UF techniques coupled with more performing analytical techniques, such as UHPLC-MS/MS. Xu et al. analyzed unbound VPA using an Agilent 1290 Ultrahigh performance liquid chromatography coupled to an AB Sciex QTRAP 4500 mass spectrometer with electrospray ionization [70]. Plasma samples were subjected to UF with an Amicon centrifugal filter device for 15 min. Optimal outcomes were obtained and the method was fully validated, showing good linearity in the range of 0.2–25 μg/mL, intra-day and inter-day precisions within ±15%, excellent recovery with no significant NSA to ultrafiltrate membranes, and an acceptable matrix effect. The method was used to quantify the unbound concentration of VPA in plasma samples obtained from 489 epileptic children receiving VPA in either mono-or polytherapy. The simple preparation, speed of separation, and reproducibility of analysis were the most interesting characteristics of this method [70].

Other methods, like UC and solid-phase micro-extraction (SPME), do not require membranes for the separation of the unbound fraction and seem not to be affected by the risk of artifacts [38]. Briefly, in the UC technique, a solution consisting of drug and proteins is placed in a centrifugal field and undergoes repeated centrifugations; this leads to the formation of a compact protein “pellet” at the bottom of the centrifugation tube that can be separated from the protein-free fraction supernatant. Although the method is very simple and does not suffer from NSA, it is worth pointing out that any protein contamination in the protein-free fraction may lead to an erroneously higher free drug concentration [62]. Nevertheless, comparative studies with different types of drugs have revealed quantitative discrepancies between the results obtained by ED and UC [71]. SPME can be considered an orthogonal approach to the previous ones, since it does not use a purely physical method for the separation of the different drug fractions, exploiting instead the different affinity for an immobilized matrix of the free drug compared to the one bound to proteins. It involves extraction of target analytes from the sample matrix via adsorption or absorption through an extracting phase coated on silica fiber or some metallic support. This is a simple and reliable method for the evaluation of additional parameters, such as binding constants, and there is an increasing interest in its use in clinical samples [72].

However, despite the continuous evolution of increasingly efficient techniques, the methodologies used for the separation of unbound fractions have not been standardized yet. The selection of the most appropriate method not only depends on the results obtained but also on the feasibility of the procedure, which can clearly contribute to a marked increase in the percentage error when measuring low unbound concentrations.

## 3. Examples of Protein Binding Influence in ASMs Polytherapeutic Regimens: Valproic Acid, Phenytoin, Perampanel, and Carbamazepine

The concomitant intake of ASMs can alter their own metabolism and modify the free plasma concentrations. This is due both to their CYP P450 inducing/inhibiting action and their potential for modifying plasma protein binding. VPA is one of the most widely used drugs in co-administration for the treatment of epileptic seizures. PHT and other drugs can displace plasma protein binding, resulting in an increase in the free VPA fraction [73]. PHT can also undergo modifications in its free fraction based on the amount of proteins, especially albumin, present in the plasma [74]. PRP is the progenitor of a new class of antiepileptics and its plasmatic free amount can be significantly affected by co-administration with other ASMs that compete for plasma protein binding [75]. CBZ metabolism is influenced by multiple factors and its plasma amount shows a non-linear correlation with the administered dose [76]. In view of the above, we report in detail some recent studies that have focused their attention on the importance of evaluating the free drug plasma concentration of these drugs.

### 3.1. Valproic Acid

Valproic acid (VPA, 2-propylpentanoic acid) is an ASM and mood stabilizer drug approved by the Food and Drug Administration in 1979, and is among the most widely used drugs for the treatment of both partial and generalized seizures in adults and children. Typically, the dosage of this drug is adjusted to achieve total serum concentrations of 50–100 µg/mL during the treatment of seizures, and 50–125 µg/mL for behaviour disorder [77]. However, several parameters can alter the plasma concentration of VPA. Although individual pharmacogenetic factors have been supposed to be involved in individual sensitivity to VPA, evidence in the literature suggests that pharmacokinetic variables play a major role in the efficacy profile of this drug [78]. Therefore, TDM is recommended during treatment with VPA, and measurement of total serum concentration has been widely used.

Valproic acid is highly bound to plasma proteins, such as albumin (≥90%), and the free fraction, which is responsible for its pharmacological and toxic effects, is between 5–10% of the total drug [79]. Renal filtration of VPA depends on both intrinsic hepatic clearance and free fraction of the drug; as the free fraction increases, drug metabolism increases, potentially leading to total serum concentrations lower than expected [80]. Therefore, monitoring VPA free fraction has been increasingly recommended in recent years [81]. Riker and co-workers monitored the free serum concentration of VPA in patients admitted to intensive care units (ICU) [82]. They observed that the protein binding of VPA was highly variable in this cohort of patients. Besides, the measured total amounts of the drug were not consistent with free fraction concentrations, even when values were corrected for albuminemia. The administration of increasing amounts of VPA seemed to lead to higher free fractions of the drug, despite a low total concentration. Despite data reported in the literature, no patients displayed a free fraction of VPA between 5 and 10%, indicating that this range was not actually predictive, at least for patients admitted to the ICU. Additionally, 4 out of 5 patients not showing adverse drug reactions had the highest free drug concentrations reported in this study, thus suggesting that the occurrence of undesired effects should also depend on further causes. However, this study suffered from some significant limitations: the subjects were mainly male, with a wide age distribution, and their numbers were too low to be statistically significant. The data also came from a single hospital, and many patients underwent administration of other drugs prior to admission (53%). Only patients with a VPA dosage in the range 50–125 µg/mL were included in this study [82]. However, the results discussed in this study should not be underestimated, since VPA is one of the AEDs preferentially administered for seizure control in ICUs.

Several variables can alter the binding of VPA to plasma proteins; free fatty acids, for example, can replace hydrophobic drugs interacting with albumin due to their high binding affinity for that protein; stearic, palmitic, oleic, and linoleic acids have been shown to increase the free fraction of VPA in a concentration-dependent manner by between 19 and 118% [83]. Moreover, concomitant administration of non-steroidal anti-inflammatory drugs, such as ibuprofen, can affect VPA binding to proteins, resulting in inappropriate dose management [84]. Physicians often prefer multiple therapies, even in the absence of data on their risk: benefit ratio. Furthermore, antiepileptic-antidepressant combinations are frequently used in patients with anxiety disorder, pain, and migraines. Particular attention should be paid to VPA when concomitantly administered with antipsychotic medications, since VPA is a mild dose-dependent enzyme inducer of these drugs [85]. Several studies have therefore dealt with this issue but, unfortunately, they were often carried out on a small number of samples thus hampering an accurate overall assessment of the management of patients undergoing treatment with VPA, especially when this was co-administered or used in patients with severe conditions.

### 3.2. Phenytoin

The use of PHT to control seizures has always been a challenge because of its narrow therapeutic range. Therefore, PHT inappropriate dosage in the management of the epileptic patients are frequently reported [86,87]. The difficulty of PHT dosage is due to its non-linear pharmacokinetics, zero-order elimination, and diverse drug–drug interactions. Therefore, drug plasmatic levels need to be constantly monitored. Plasma protein binding by PHT is also relevant, and only the free portion of the drug, consisting of about 10% of the total plasma concentration, results in a therapeutic effect. The therapeutic reference range for this AED, concerning its total plasma concentration, is estimated to be 10–20 µg/mL. Considering this, the Sheiner–Tozer equation has historically been used to adjust the detected PHT concentration, taking into account albumin levels [88]:

1 $$\mathrm{Predicted}\;\mathrm{free}\;\mathrm{PHT}=\frac{\mathrm{Measured}\;\mathrm{total}\;\mathrm{PHT}}{\left(\mathrm{Albumin}*0.1\right)+0.1}\;\times \;0.1$$

The work of Krasowski and co-workers showed how the measurement of free PHT could be adjusted using the Sheiner–Tozer equation [73]. However, this method suffers of a wide dispersion of data and the results obtained are not always congruent with the clinical interpretation of the remission of epileptic seizures. It was also highlighted how the variation in total PHT is related to abnormally low levels of plasmatic albumin (hypoalbuminemia) that led to an increase in free PHT, although other factors such as uremia or interactions with other ASMs (e.g., VPA) could be involved [73,74]. Consequently, several authors tried to use modified versions of the Sheiner–Tozer equation to overcome these biases [74]. The relationship between free and total PHT has been well described as inconsistent by Jun and coworkers, who have tested different mathematical binding models with discordant results [89]. First, the authors demonstrated that the Tozer model described above was not consistent with clinical data; therefore, they used other mathematical models, concluding that the linear binding model was the one that best described PHT binding under normal clinical conditions. Conversely, in patients treated with high concentrations of PHT, the single-site non-linear model provided results that were more accurate. However, all these results were influenced by albumin concentration [89]. Like many others, the limit highlighted by this study was that the data collected during the early post-administration periods was insufficient, making it impossible to accurately estimate the absorption process in the proposed models. Moreover, the number of samples considered was too small to give a statistical validation of the predictive model. A recent work by Barra et al. reached the same conclusions, showing that the Sheiner–Tozer, as well as equation derived from it, underestimated the real levels of free PHT in patients admitted to ICUs [90]. This study also underlined that the error in the quantitation of free drug was one order of magnitude greater in critically ill patients than in those admitted to a non-critical unit. This bias made it impossible to detect supra-therapeutic levels in critically ill patients, possibly because of the physiological changes occurring during acute illness (e.g., hypoalbuminemia, liver or kidney failure, uremia, and competition with other plasma protein-bound drugs). All these variables can lead to higher-than-expected free PHT fractions. The main limitation of this study probably was that, being retrospective, it did not indicate which levels of free PHT had been measured during the worsening of patient conditions and which ones during routine screening. Furthermore, the data considered in this research differed from those reported elsewhere, because the immunoassays of PHT were performed at 25 °C, while in many other laboratories they are carried out at 37 °C.

Several studies conclude that, given the inefficiency to detect supra-therapeutic PHT levels because of large errors found with traditional equations, therapeutic monitoring of free PHT should always be performed. Further investigations are needed to compute a predictive formula that better describes the drug absorption model than traditional Winter–Tozer or other derived equations, especially in the clinical management of a critically ill patient.

### 3.3. Perampanel

Perampanel is a selective non-competitive antagonist of the AMPA glutamate receptor, a new class of ASMs, approved by the FDA for the adjunctive treatment of partial seizures with or without generalized disease in patients with epilepsy aged ≥12 years [91]. PRP is taken orally and undergoes liver metabolism via CYP3A4 and CYP3A5 enzymes [92]. It is initially administered at a dosage of 2 mg/day, with sequential increments of 2 mg up to 8 mg/day. The higher dosage, 12 mg/day, is restricted to those patients whose tolerability at 8 mg/day has already been demonstrated. Monitoring PRP plasma levels prior to dosage increase is always recommended. PRP is over 95% bound to proteins, mainly albumin, therefore the serum free drug fraction is quite low (approximately <5%) [93]. The high affinity of PRP towards plasma proteins is probably the cause of the slow hepatic metabolism of this drug, resulting in a negligible first pass effect and an unusually long half-life. Although its metabolites are inactive, and up to 70% of them are excreted in faeces, they can bind to plasma proteins causing idiosyncratic toxicity, including hepatotoxicity, especially in the presence of low glutathione levels [94]. Attention in the management of epileptic patients must be paid to the effect of eventual co-administered enzyme-inducing drugs, which may increase PRP clearance. Co-administration of CBZ, PHT, OXC, and, to a lesser extent, TPM can result in a significant decrease (up to 67%) in PRP clearance [29,31]. VPA can also affect PRP kinetics, increasing PRP plasma concentration in proportion to the dose [32].

On the other hand, PRP is itself a weak enzyme inducer and could alter the metabolism of other co-administered drugs. However, many drug interactions involving PRP depend on its binding to plasma proteins; for example, OXC clearance is reduced by 26% when it is co-administered with PRP, resulting in a significant increase in drug serum levels [95]. Again, the importance of an accurate monitoring of plasma levels of PRP and co-administered drugs is evident; besides, the direct measurement of free and protein-bound fractions of PRP would be more informative and predictive of possible undesired effects or drug interferences. However, the number of studies on the effectiveness of monitoring the total plasma concentration and the measurement of free and protein-bound fractions for the optimization of therapies is still quite small. Therefore, further research is necessary in this field to set up protocols suitable to optimize PRP dosage when this drug is administered in polytherapy.

### 3.4. Carbamazepine

Carbamazepine (CBZ) is a tricyclic compound used as an anticonvulsant and mood stabilizer. This drug is mainly used in the management of epilepsy, bipolar disorder, attention deficit and schizophrenia; it is a Na^+^ and aminobutyric acid (GABA) channel inhibitor, which results in reducing neuronal excitability, and it also inhibits the reuptake of biogenic amines [96]. CBZ is largely bound to plasma proteins, and most of the drug enters the bloodstream from tissue reserves; therefore, this drug displays a high distribution volume. Nevertheless, the half-life of CBZ after the first administration is approximately 30 h; it is indeed converted by CYP 3A4 and CYP2C8 to its active metabolite carbamazepine-10,11-epoxide (CBZ-E), and further less-active derivatives such as dihydroxy-epoxide products. Following subsequent administration, a faster elimination occurs due to the self-inductive effect of CBZ on cytochrome P450 [97]. Several studies have shown that the metabolism of some drugs can be affected when they are co-administrated with CBZ. On the other hand, CBZ plasmatic levels increase when it is co-administered with serotonin reuptake inhibitors [98].

Due to the high affinity of CBZ towards plasma proteins, the free and protein-bound fraction of the drug and its active metabolite must be taken into account when TDM studies are carried out. A paper from Gao et al. reported a high inter-individual variability of the different fractions of CBZ, regardless of the administered dose [69]. This study was based on the use of an innovative approach aimed at simultaneously quantifying the free and protein bound CBZ fraction in plasma [69]. According to the authors, this analytical method had to be simpler to apply than those previously described [67], however results obtained highlighted that its use to perform dose adjustments was not effective in clinical practice. The work highlights the importance of monitoring the permanence in plasma of CBZ and its metabolite CBZ-E due to its neurological and motor toxicity in humans. The main limitations of the study were the use of animal models and the evaluation of the pharmacokinetic parameters of the drug only at the mean tolerated dose of 1.4 mmol / kg for ethical reasons. Data collected from the literature agreed on the importance of measuring CBZ and its metabolites and underlined the necessity of considering the difference between the free and bound fraction of the drug. It is also desirable to define a suitable equation for the dosage adjustment in therapy.

## 4. Saliva as an Alternative Fluid for Measuring the Antiseizure Medications Free Fraction

As underlined above, monitoring the free fraction of antiseizure drugs may be tedious and time consuming. Moreover, for some ASMs, or under certain analytical conditions, measured drug concentrations may not reflect the actual ones. Plasma drug concentrations are variable between arteries, which more or less have the same drug level, and veins that show instead very variable drug levels. Veins coming from different organs may have different drug concentrations. Immediately after drug input, arterial drug concentration is higher compared to the corresponding venous compartment. The opposite phenomenon is observed when a drug is being actively eliminated from the body. All these aspects may shed light on discrepancies existing between plasma venous concentrations and drug efficacy. From a purely theoretical point of view, to evaluate the efficacy of a drug specifically directed towards brain cells, such as antiseizure drugs, it would be necessary to measure its concentration in the specific district, i.e., in the brain interstitial fluid. The amount of drug present in the brain interstitial fluid corresponds to the free fraction of the drug and is the one that effectively exerts the pharmacological effect. It goes without saying that this approach is neither possible nor ethical, therefore, when possible, the free fraction is obviously measured on plasma samples.

A promising alternative to plasma/serum or whole blood sampling is the use of saliva. During the 1970s, saliva was first investigated as an alternative biological fluid to monitor CBZ, PHT, and VPA [99,100,101,102,103]. In the last decade, saliva has been reconsidered as a useful fluid to monitor several ASMs and their free fractions [104,105]. Compared to plasma/blood monitoring, saliva may show some advantages. First, for many drugs, saliva concentration may reflect the actual free fraction in serum (the active drug fraction). Saliva can be collected in a simple and non-invasive manner, avoiding most of the drawbacks related to phlebotomy; indeed patients generally prefer saliva sampling to blood sampling. To collect saliva there is no need for trained personnel and sampling can be performed “at home”, in a more comfortable environment for both patients and their caregivers. Samples can be collected at any time needed (i.e., trough and pre-dose) and sent to the laboratory, thus facilitating pharmacokinetic studies.

Some disadvantages include a possible contamination by the ingested drug (which can easily be avoided by sampling right before drug ingestion), low sample volume, or high density of saliva (both issues can be solved in the laboratory by adding a small amount of saline solution or buffer to the collected sample). ASMs concentration may be very low in saliva, thus specific analytical methods should be available or should be validated in the laboratory.

Saliva is produced in the salivary glands by ultrafiltration of arterial plasma in the so-called acini cells, and is made up of water, electrolytes, and proteins mixed with contaminating cells and bacteria and its composition varies along the way from the acini cells to the collecting ducts. In the ducts, sodium is actively reabsorbed, potassium and protons secreted, and large amounts of bicarbonate ion reabsorbed. These differences in saliva composition will create differences in pH and ionic strength that in turn may affect free drug concentration [106,107].

When saliva is secreted into the oral cavity, drug concentration and pH differ significantly from the values they have in the upper zone of the ducts. Saliva becomes more acidic and ASMs concentration may vary depending on their physicochemical characteristics. The volume recovered determines whether the concentration of the drug in this fluid would be closer to the free plasma venous value or to the arterial one. For non-ionized ASMs, the smaller the saliva volume (usually obtained without stimulation, or the first fraction obtained after stimulation), the closer it is to the free plasma venous concentration. In the case of weak acid molecules, lower values than the corresponding free values in the venous plasma are obtained because the pH in the lower part of the ducts is more acidic compared to the blood (and the acinus). In contrast, in the case of basic antiseizure drugs, a value above the free plasma venous concentration should be expected.

In large saliva volumes or in saliva samples obtained by stimulation (chewing gum or placing small amounts of citric acid crystals on the tongue), ASMs concentrations approach those of the top of the ducts (acini). As reported in the literature [108,109], drug concentration variability in saliva might be reduced by using stimulated saliva sampling. For several ASMs, mainly those that are lipophilic and not ionized at the salivary pH range (i.e., PHT, CBZ and PHT), stimulated or unstimulated saliva concentrations highly correlated with plasma concentrations. It is also interesting to note that, regardless of the time, after the dose, during the uptake, or elimination phase, the drug level in stimulated saliva (SS) is always related to the serum free level in arterial plasma.

Perhaps the best advantage of saliva sampling is that it allows a very simple, specific, and sensitive test for monitoring ASMs free fraction, given the current availability of clinical LC-MS/MS techniques in TDM laboratories. In most cases, it is possible to prepare samples with a very simple “dilute and shot” method, avoiding concentration and precipitation steps while injecting a diluted saliva sample without affecting method performances [110]. Moreover, the sensitivity of LC-MS/MS also allows the examination of samples based on a dried saliva spot (DSS). These devices, similarly to dried blood spot (DBS) or dried plasma spot (DPS) [111] can be used to collect desiccated saliva and can easily be handled and shipped to the reference laboratory [112,113,114,115].

As shown in the table below, saliva can be used for monitoring either total or free fraction of some ASMs (Table 2). In this section, we will consider only those drugs that may need the monitoring of plasma free fraction discussed in Section 3.

The physicochemical properties of VPA (i.e., a weak acid with a pKa of 4.9), and the pH gradient between serum and saliva result in only small quantities of VPA passing into saliva; furthermore, concentrations are widely distributed. Stimulation by citric acid did not increase the low saliva VPA concentrations, and the correlation between saliva and serum VPA concentrations (both total and free) was poor [122,148]. Since salivary VPA concentrations poorly correlate with total and free serum VPA concentrations [122,136,148,149,150,151], saliva cannot be used as an alternative matrix for the TDM of VPA [104].

There have been many studies investigating the distribution of PHT in saliva and the relationship between saliva PHT concentration and both serum total and serum free PHT concentrations in both adults and children with epilepsy [116,121,122,123,124,131,136,137,138,139,140,141,142,143,144,145,146]. PHT distributes into saliva reaching a concentration that is similar to the free drug concentration in serum. Saliva PHT concentrations and both serum total PHT (r2 = 0.85–0.99) and serum-free PHT (r2 = 0.96–0.99) concentrations are significantly correlated. There is some evidence that PHT distribution in saliva depends on whether resting saliva, stimulated saliva, or reduced flow saliva is collected. For the latter two situations, PHT concentrations in saliva are decreased and increased, respectively [147]. Thus, unstimulated saliva should be used, and this provides a useful alternative matrix for TDM of PHT [104].

PRP is over 95% bound to proteins, mainly albumin, therefore its serum free drug fraction is quite low (approximately <5%). Recently, Kim et al. [152] have used saliva for the TDM of PRP. The authors reported that a linear relationship is present between the total PRP concentrations in saliva and both the total and free PRP concentrations in plasma (both *p* <0.001; r = 0.678 and r = 0.619, respectively). The modification in PRP concentration caused by the CYP3A4 inducer did not affect the correlation between saliva and plasma concentrations (all *p* < 0.01). These results demonstrate that, in the presence of a suitable and sensitive analytical method, saliva can be used as an alternative matrix for the TDM of PRP free fraction.

CBZ has a half-life varying between 8 to 20 h in patients on chronic therapy and may be greater after a single dose due to metabolic autoinduction, whereas the half-life of the active metabolite CBZ-E is around 34 h. CBZ and CBZ-E are both protein-bound with different percentages: 75% for CBZ and 50–60% for CBZ-E [110]. Concentrations of CBZ and CBZ-E in saliva are significantly correlated with both serum total (r2 = 0.84–0.99 and r2 = 0.76–0.88, respectively) and serum-free concentrations (r2 = 0.91–0.99 and r2 = 0.75–0.98, respectively). Thus, saliva may be used as an alternative matrix for TDM of both CBZ and CBZ-E [104] (Table 2).

## 5. Conclusions

The relevance of drug–plasma–protein interactions in determining the bioavailability of therapeutic agents is well known. Evidence of this is, for example, the ever-increasing number of drugs covalently conjugated to proteins that are currently available on the market [153]. A drug linked to a polypeptide, in fact, remains in the bloodstream for a much longer time than the free drug, thus escaping many metabolism and elimination processes. On the other hand, a protein-bound compound is not able to interact with its biological target(s), in particular in the case of drugs whose activity is directed towards body compartments that are not accessible to large hydrophilic molecules. When designing a therapeutic plan, it is therefore important to take into due consideration the affinity of the different drugs for plasma proteins to achieve an optimized pharmacokinetics. Noteworthy, the effective amount of a drug interacting with albumin and other blood proteins as well as the resulting drug/protein complex dissociation kinetics depend on multiple factors, which include the different concentration levels of the various protein species involved and their binding with other endogenous (for example lipids) or exogenous (such as other drugs) molecules.

All these aspects, which are often difficult to predict and may be affected by inter-individual variability, do not always determine important effects on the efficacy and safety of several drugs [154]. However, when dealing with bioactive molecules with a narrow therapeutic window and/or administered in co-therapy, such as many ASM medications, an altered and/or unpredictable binding with soluble proteins may be of great concern due to either lack of efficacy of the treatment or occurrence of adverse effects [155].The aim of this review was to present some recent research focused on the evaluation of the free/protein-bound ratio of different ASMs such as VPA, PHT, PRP and CBZ, and to discuss the analytical approaches developed so far and the clinical significance of these analyses. Several methodologies, as well as different biological matrixes, are currently available that potentially allow for the TDM of unbound forms of several ASMs. Nevertheless, a limited number of studies, often retrospective, have so far linked real-world clinical context pharmacokinetic monitoring to clinical outcomes or neurologic adverse effects. Moreover, the small sample size often considered in these clinical studies represents an additional and serious drawback. Prospective validation studies are undoubtedly needed, which would help unravel the link between free drug plasma concentration and the efficacy of the therapeutic regimen used, also allowing us to better define therapeutic concentration ranges with respect to the occurrence of adverse effects.

## Figures and Tables

**Table 1:** Examples of highly bound ASMs.

Drug	Target Molecule	% Bound	Plasma Levels/Therapeutic Range	Interactions
phenytoin	Voltage-dependent neuronal Sodium ion channels [11]	90 [11]	10–20μg/mL(total) [11,17]. 1–2μg/mL (free) [17]	Induces: CYP3A4, CYP2C9, CYP2C19, CYP1A2, UGT [11,17] and interacts with many drugs, including other ASMs.
valproic acid	Elevation of brain GABAergic; Block of Sodium ion channels [11]	90 [11]	50–100 μg/mL(total) [11,17] 5–12.5 μg/mL (free) [17]	Inhibits: CYP2C9, UGTs, Epoxide hydrolase [17]. Decrease lamotrigine clearance increasing its concentration Low decrease of PMP clearance
phenobarbital	Chloride ion channels within GABA_A_ receptors [11]	60 [11] 55 [17]	10–30 μg/mL [11] 15–40 μg/mL [17]	Induces: CYP3A4, CYP2C9, CYP2C19, CYP1A2, CYP2E1, UGT. May increase the clearance of molecules and drugs that are co-administered [11,17] and interacts with many drugs, including ASMs.
carbamazepine	Voltage-dependent Sodium ion channels in cell membranes [11]	~75 [11] 76 [17]	4–12 μg/mL (total) [11,17]	Converted to a biologically active epoxide metabolite. Induces: CYP3A4, CYP1A2, CYP2B6, CYP2C9, CYP2C19, P-glycoprotein, UGT [11,17] and interacts with many drugs, including ASMs
perampanel	Non competitive AMPA glutamate receptor antagonist [1,18,30]	96 [18] 95 [17]	0.2–1 μg/mL (total) [27]	Induces: CYP3A4 (weak) [17] CBZ, PHT, and PB decrease PRP concentration [31] VPA can slightly increase PRP concentration [32]
tiagabine	inhibits GABA reuptake at the synapse [9]	96 [17,18]	0.02–0.2 μg/mL [18,27]	---
stiripentol	Increase of brain γ-aminobutyric acid (GABA) levels by interferingwith GABA uptake and metabolism [18]	99 [18]	4–22 μg/mL [18,27,33,34,35]	Inhibits many CYPs (CYP3A4, 1A2, 2C19) and interacts with many drugs, including ASMs [18]. Decreases PB and PHT concentrations [36]

**Table 2:** Comparison between saliva and plasma free fraction monitoring.

ASMs	Plasma Protein Binding (%)	Monitoring Useful of:		Saliva Monitoring References
		Saliva	Plasma Free Fraction	
CBZ	75	**yes**	**yes**	[104,116,117,118,119,120,121,122,123,124,125,126,127,128,129,130,131,132,133,134,135,136]
PHT	92	**yes**	**yes**	[104,118,122,123,124,137,138,139,140,141,142,143,144,145,146,147,148]
VPA	74–93	**n.a. (see text)**	**yes**	[104,124,136,148,149,150,151]
PRP	98	**yes**	**yes**	[104,105,106,107,108,109,110,111,112,113,114,115,116,117,118,119,120,121,122,123,124,125,126,127,128,129,130,131,132,133,134,135,136,137,138,139,140,141,142,143,144,145,146,147,148,149,150,151,152]

n.a. not applicable.

## Data Availability

Not applicable.
